# Massively Convergent Evolution for Ribosomal Protein Gene Content in Plastid and Mitochondrial Genomes

**DOI:** 10.1093/gbe/evt181

**Published:** 2013-11-19

**Authors:** Uwe-G Maier, Stefan Zauner, Christian Woehle, Kathrin Bolte, Franziska Hempel, John F. Allen, William F. Martin

**Affiliations:** ^1^LOEWE Centre for Synthetic Microbiology (SYNMIKRO), Philipps-Universität, Marburg, Germany; ^2^Laboratory for Cell Biology, Philipps-Universität, Marburg, Germany; ^3^Institute of Molecular Evolution, Heinrich-Heine-Universität, Düsseldorf, Germany; ^4^School of Biological and Chemical Sciences, Queen Mary University of London, London, United Kingdom

**Keywords:** mitochondria, plastids, organelle genomes, ribosomal proteins, evolution, gene transfer

## Abstract

Plastid and mitochondrial genomes have undergone parallel evolution to encode the same functional set of genes. These encode conserved protein components of the electron transport chain in their respective bioenergetic membranes and genes for the ribosomes that express them. This highly convergent aspect of organelle genome evolution is partly explained by the redox regulation hypothesis, which predicts a separate plastid or mitochondrial location for genes encoding bioenergetic membrane proteins of either photosynthesis or respiration. Here we show that convergence in organelle genome evolution is far stronger than previously recognized, because the same set of genes for ribosomal proteins is independently retained by both plastid and mitochondrial genomes. A hitherto unrecognized selective pressure retains genes for the same ribosomal proteins in both organelles. On the *Escherichia coli* ribosome assembly map, the retained proteins are implicated in 30S and 50S ribosomal subunit assembly and initial rRNA binding. We suggest that ribosomal assembly imposes functional constraints that govern the retention of ribosomal protein coding genes in organelles. These constraints are subordinate to redox regulation for electron transport chain components, which anchor the ribosome to the organelle genome in the first place. As organelle genomes undergo reduction, the rRNAs also become smaller. Below size thresholds of approximately 1,300 nucleotides (16S rRNA) and 2,100 nucleotides (26S rRNA), all ribosomal protein coding genes are lost from organelles, while electron transport chain components remain organelle encoded as long as the organelles use redox chemistry to generate a proton motive force.

## Introduction

Plastids arose from cyanobacteria, mitochondria arose from proteobacteria, and both organelles have retained genomes, the sequence and structure of which unmistakably betray their prokaryotic origin (Gray et al. 1999; Stoebe and Kowallik 1999). However, the genomes of both organelles are highly reduced relative to those of their free-living cousins, whose genomes often exceed 5,000 genes (Timmis et al. 2004). Photosynthetically active plastids—chloroplasts—encode between ∼80 proteins in land plants and ∼200 in red algal lineages (Allen et al. 2011). Mitochondria that harbor a respiratory chain encode between 3 and 63 protein coding genes, the most gene-rich mitochondrial genome being found in the jacobid *Reclinomonas* (Lang et al. 1997), and the smallest mitochondrial genome being found in the malaria parasite *Plasmodium* (Joseph et al. 1989). Despite these massive genome reductions, both plastids and mitochondria contain more than 1,000 proteins that underpin their primarily prokaryotic biochemistry. The great majority of these proteins are synthesized in the cytosol and imported, as precursors, for processing into their mature forms, and the genes for many of these imported proteins were transferred from the organelle to the nucleus during the course of evolution (Martin et al. 2002). Eukaryotic genome sequences reveal that gene transfer to the nucleus is an ongoing evolutionary process. Among the largest DNA segments that have been transferred from organelles are a complete 367 kb mitochondrial genome in the *Arabidopsis* nucleus (Stupar et al. 2001) and a complete 131 kb chloroplast genome in the rice nucleus (Huang et al. 2005). Such examples demonstrate that the process underlying organelle gene relocation to the nucleus is incorporation of bulk organelle chromosomes, most probably stemming from lysed organelles (Cavalier-Smith 2010) with the biochemical mechanism of organelle DNA integration having been identified as nonhomologous end joining at double-strand breaks (Hazkani-Covo and Covo 2008; Hazkani-Covo et al. 2010). Consistent with this view, the frequency with which organelle DNA integrates into the tobacco nuclear genome via double-strand break repair is increased under physiological stress (Wang et al. 2012).

Transfer of organelle DNA is thus commonplace and widespread, with nearly all investigated lineages revealing abundant, recently inserted segments of organelle DNA in chromosomes of the cell nucleus (Bensasson et al. 2001; Kleine et al. 2009; Hazkani-Covo et al. 2010). The exceptions, lacking recent transfers, are lineages that harbor only one organelle per cell, because this single organelle has to be retained for viability and inheritance (Lister et al. 2003), and lineages, such as trichomonads, that have lost organelle genomes completely (de Paula et al. 2012). Moreover, a number of evolutionary pressures are known that—in theory—strongly favor nuclear over organelle localization of genes. These include the mutagenic nature of reactive oxygen species that arise from the electron transport chains of mitochondria and chloroplasts (Allen and Raven 1996), population genetic aspects in animal mitochondria (Lynch et al. 2006), and the physical polarity of endosymbiosis, which creates a one-way street of gene transfer from lysed organelles to the host (Doolittle 1998; Martin and Herrmann 1998). Clearly, there are no sequence-specific barriers to DNA transfer from an organelle to the nucleus, as indicated by analyses of mitochondrial and plastid DNA fragments in nuclear chromosomes (Timmis et al. 2004; Kleine et al. 2009) and by experimental work demonstrating gene transfer from transformed mitochondria (Thorsness and Fox 1990) and plastids (Huang et al. 2003; Wang et al. 2012). Thus, there are ample reasons, there has been sufficient time, and there have been countless opportunities during evolution to relocate all organelle genes to the nucleus. In utter defiance of these pressures, nature has nonetheless tenaciously retained genes in organelles. There must be an overarching selective pressure that overrides such mutational, population genetic, and physical orientation forces (Race et al. 1999). What anchors DNA in organelles?

Of the various theories put forward to account for the retention of organelle genomes (reviewed in Allen 2003; Barbrook et al. 2006), only one explains the staggering degree to which mitochondria and plastids have undergone massively parallel evolution to converge upon the same functional gene set in all eukaryotes: key components of the photosynthetic electron transport chain in thylakoids and of the respiratory chain in the mitochondrial inner membrane. The colocation for redox regulation (CoRR) hypothesis (Allen 1993, 2003) posits that genes remain in organelles because individual organelles need to regulate the assembly and stoichiometry of the components in their membrane-associated electron transport chains (Allen et al. 2005). Failure to adjust the stoichiometry of those components rapidly leads to redox imbalance, energetic losses, an overreduced or underreduced quinone pool, and hence to the nonenzymatic transfer of single electrons from semiquinones to O_2_-producing reactive oxygen species, oxidative stress, and, ultimately, organelle and cell death. This can be illustrated with a simple scenario in a plant cell harboring about 100 plastids: had all genes for the photosynthetic electron transport chain been moved to the nucleus and were one plastid to require more photosystem I, for example, to maintain redox balance, then this plastid could signal to the nucleus, but the nucleus would be able to respond only by increasing photosystem I synthesis generally, that is, for all of the plastids in the cell. The remaining 99 plastids, which were initially fine, would then be out of redox balance, requiring more photosystem II and less photosystem I. Thus, an individual organelle needs to be able to sense and regulate the redox state of its own bioenergetic membranes. This hypothesis both demands and predicts the presence of proteins that sense the redox state of the quinone pool to allow individual and specific plastid gene regulation (Puthiyaveetil et al. 2010). Such plastid redox–sensor proteins have been found (Puthiyaveetil et al. 2008; Puthiyaveetil and Allen 2009) and shown (Puthiyaveetil et al. 2013) to be required for photosynthetic, redox control of plastid transcription (Pfannschmidt et al. 1999). Similar reasoning applies to the respiratory chain complexes in mitochondria although the corresponding redox sensors have not yet been identified (de Paula et al. 2012).

Thus, CoRR directly accounts for the observation that plastid and mitochondrial genomes have come to retain exactly the same kind of protein-coding genes: essential components of the photosynthetic and respiratory electron transport chain and of the ribosome needed to express them in the organelle. But among the ribosomal proteins (r-proteins), there is the hitherto unnoticed circumstance that genes for some r-proteins have a far greater tendency to be retained within the organelle than others. Here we report the distribution of ribosomal protein-coding genes in chloroplast and mitochondrial genomes. We observe that in multiple independent eukaryotic lineages the genomes of both organelles tend to retain the same core set of ribosomal protein-coding genes. This observation uncovers a case of massively convergent evolution and provides hints concerning the selective pressure that produces it.

## Materials and Methods

A data set containing more than 300 plastid genomes available at the genome section (http://www.ncbi.nlm.nih.gov/genomes/GenomesGroup.cgi?taxid=2759&opt=plastid, last accessed December 2, 2013) of the NCBI server (March 2013) was examined with respect to genes encoding r-proteins using standard text searching and sequence searching methods (Blast) in a UNIX environment. r-Proteins in mitochondrial genomes and in genomes of bacterial endosymbionts (McCutcheon 2010) were identified in the same way.

## Results and Discussion

### The Plastid Core

**Fig. 1.—:**
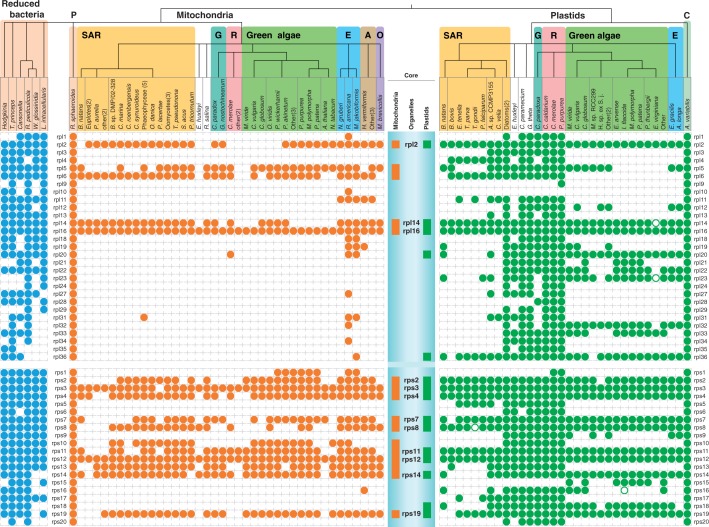
Presence/absence pattern of organellar-encoded r-proteins in eukaryotic groups. Filled circles indicate presence (green = plastid; orange = mitochondrion) of specific proteins, while empty circles represent pseudogenes. The core genes of mitochondria, plastids, and their shared set are represented by squares or protein names shown in the blue box. Accession numbers and full species names are given in supplementary table S1, Supplementary Material online. For comparison, the r-protein gene content of several sequenced endosymbiotic bacteria (McCutcheon 2010) is shown in the left panel. Note that there are no organelle encoded copies of *rpl*15, *rpl*17, *rpl*25, *rpl*30, or *rps*21 that we could detect in any lineage. Because these r-proteins are missing in all chloroplast and mitochondrial genomes surveyed here, they were not included in the figure. There are, however, many nuclear-encoded copies of these five prokaryotic genes in the databases. Organisms with identical patterns and belonging to the same taxonomic group were merged with a following number indicating their frequency. The tree is based on Katz (2012). Abbreviations of taxonomic groups: A = Amoebozoa; E = Excavata; G = Glaucophytes; O = Opisthokonta; R = Red algae; P = proteobacteria; C = cyanobacteria; SAR = stramenopiles, alveolates and rhizaria.

Using the 12 sequenced genomes of photosynthetically active plastids available in 1999, Stoebe and Kowallik (1999) identified a ribosomal gene core in plastids encompassing the chlorophyte, rhodophyte, and glaucocystophyte lineages. The plastid ribosomal gene core encompasses the genes for the 30S ribosomal subunit proteins Rps2, Rps3, Rps4, Rps7, Rps8, Rps11, Rps12, Rps14, Rps18, Rps19 and for the 50S subunit proteins Rpl2, Rpl14, Rpl16, Rpl20, and Rpl36. Using a much larger sample, we find that the set of genes for r-proteins common to all plastids and as determined by Stoebe and Kowallik (1999), which we call the plastid ribosomal gene core, is, in principle, still intact. Minimal variations from this common ribosomal gene core have been detected in apicoplasts, the reduced, nonphotosynthetic plastids of apicomplexan organisms, which encode the ribosomal gene core lacking rps14, rps18, and rpl20 (Wilson and Williamson 1997). Other parasitic, photosynthetic inactive organisms, such as *Epiphagus virginania* (Wolfe et al. 1992) or the parasitic green alga *Helicosporidium* sp. (de Koning and Keeling 2006) encode a slightly reduced version of the ribosomal gene core in their plastid genome (fig. 1).

Parasitic life style does not necessarily lead to losses of genes of the ribosomal core as shown by some *Cuscuta* species (Funk et al. 2007; McNeal et al. 2007), which are only partially photosynthetically active but still encode the complete ribosomal gene core. The plastid ribosomal gene core is present in the nonphotosynthetic alga *Cryptomonas paramecium* (Donaher et al. 2009) as well, whereas in the nonphotosynthetic euglenoid flagellate *Astasia longa rps*18 is the only missing small subunit (SSU) ribosomal gene of the core set (Gockel and Hachtel 2000). The reasons behind the retention of a few protein coding genes, pseudogenes, and ribosomal protein genes in genomes of nonphotosynthetic plastids are still debated and might involve plastid-encoded tRNAs (Barbrook et al. 2006). Thus, only rarely are gene losses of members of the ribosomal gene core detected in plastid genomes of many independent lineages (fig. 1), and these exceptions are always nonphotosynthetic, indicating that, in photosynthetically active plastids, the members of the ribosomal gene core have to be expressed in the organelle as opposed to being imported from the cytosol. Reverse genetic analyses of some members of the core set of plastid-encoded ribosomal genes (Fleischmann et al. 2011) support this view. Redox regulation anchors ribosomes to organelles to supply bioenergetic proteins, but why are genes for some r-proteins more likely to be anchored than others?

### Chloroplast Ribosomal Proteins and Ribosome Assembly

**Fig. 2.—:**
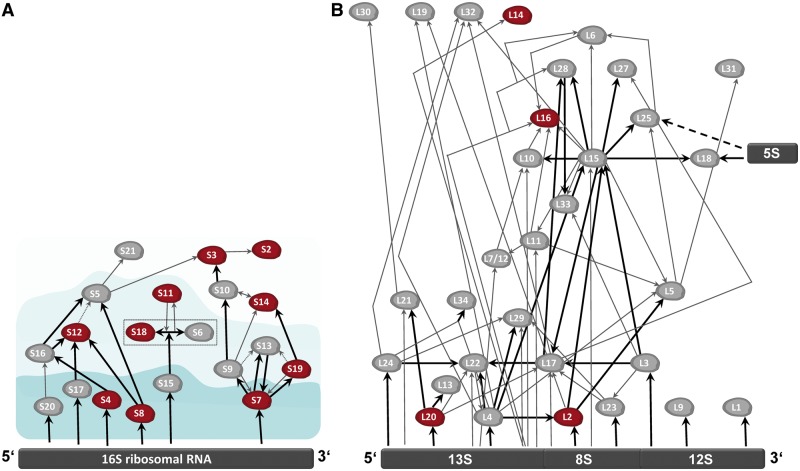
Assembly maps of 30S and 50S ribosomal subunits according to Kaczanowska and Ryden-Aulin (2007). (*A*) 30S ribosomal subunit. Red: ribosomal gene core. Areas indicate primary (dark blue), secondary (blue), and tertiary (light blue) binding proteins. Black arrows: strong dependence for binding; gray arrows: weaker dependence; dashed gray arrows: very weak dependence. Dashed box indicates a binding complex of the proteins S6 and S18. (*B*) 50S ribosomal subunit. Red: ribosomal gene core. Black arrows: strong dependence for binding; gray arrows: weaker dependence.

Prokaryotic ribosome assembly is illustrated by *E. coli* assembly maps, which indicate the temporal and spatial interactions of rRNAs and proteins during the biogenesis of ribosomal subunits (Herold and Nierhaus 1987; Nierhaus 1991; Kaczanowska and Ryden-Aulin 2007; Mulder et al. 2010; and references therein). For the small, 30S, subunit, 21 r-proteins are structural parts of this complex; many of them interact directly with the rRNA and several are involved in protein–protein interactions during assembly. Plastid ribosomes descend from cyanobacterial homologs and plastid-encoded r-proteins are broadly homologous to their bacterial counterparts (Yamaguchi and Subramanian 2000; Yamaguchi et al. 2000). Projecting the plastid-encoded ribosome protein core onto the *E. coli* assembly map (Prechtl and Maier 2001; fig. 2) reveals that seven of the ten SSU r-proteins of the plastid ribosomal gene core are involved either in direct binding to the 16S rRNA or in protein–protein interactions. For the large, 50S, plastid ribosomal subunit (LSU), a similar picture emerges, with five out of the six plastid encoded ribosomal core proteins showing direct contact with the LSU rRNA, in addition to multiple protein–protein interactions (Kaczanowska and Ryden-Aulin 2007).

The conservation of structural components underpinning plastid ribosomal assembly suggests that this process in plastids is probably not drastically different to that in *E. coli*. Accordingly, we propose that the set of r-proteins retained by plastids is determined by spatiotemporal constraints imposed by assembly of ribosomal subunits.

This proposal is not only compatible with Allen’s CoRR hypothesis for the retention of organelle genomes, it is nested within it and can be seen as a corollary thereof. The CoRR hypothesis posits that the selection pressure underlying the retention of organelle genomes is the need for redox-dependent regulation of the genes for components of the electron transport chain of bioenergetic organelles (Allen 1993, 2003). CoRR directly accounts for the observation that both plastid and mitochondrial genomes have independently converged upon exactly the same functional set of genes: components of the electron transport chain and of the ribosome that is required for protein synthesis within the organelle. Yet among the subset of organelle encoded genes for r-proteins, there is no reason to suspect that some r-proteins should be under redox regulation and others not. Accordingly, the CoRR hypothesis has never generated suggestions that such should be the case for specific r-proteins. Rather, CoRR simply demands the presence of functional ribosomes within the organelle, regardless of where the corresponding r-proteins are encoded. Yet among the r-proteins, there is a clear preference to retain some over others. Thus, although the CoRR hypothesis accounts for the retention of genes for proteins of the electron transport chain and the ribosome so that the former may be synthesized in the organelle, it makes no prediction concerning which r-proteins are preferentially retained. Ribosomal assembly appears to fill this void.

If ribosome assembly is the selective pressure behind the preferential retention of some r-proteins over others, and if ribosomal assembly is conserved from *E. coli* to plastids, then the same pattern of ribosomal genes should be conserved in mitochondria, and the ribosomal gene core of the plastid should, in principle, resemble that of the mitochondrion. This prediction is readily checked.

### Ribosomal Proteins Encoded in Mitochondrial Genomes

A survey of mitochondrial genomes for presence of ribosomal protein genes reveals a striking congruence with the plastid ribosomal core (fig. 1), and it is remarkable that this convergence has apparently not previously been noted. In the 30S subunit, mitochondrial genomes have a strong tendency to retain genes for 11 proteins: the list is virtually identical to that for the plastid ribosomal core, except that *rps*18 is missing in mitochondria and *rps*13 (present in mitochondria) is missing in the plastid core. In the 50S subunit, there is a strong tendency for mitochondria to retain genes for five r-proteins: *rpl*2, *rpl*14, *rpl*16 (which are present in the plastid ribosomal core), with *rpl*5 and *rpl*6 present in mitochondria but lacking in the plastid core, and *rpl*20 and *rpl*36 present in the plastid core but lacking mitochondria.

Mitochondria arose before plastids (Cavalier-Smith 2010; Parfrey et al. 2011) and thus have had more time to undergo genome reduction by gene transfers and gene losses. This attrition is visible in figure 1. However, our suggestion about ribosomal assembly keeping genes for some r-proteins in organelles appears at first sight to miss the mark, because the mitochondria of some organisms encode r-proteins and others do not. This will require a corollary, described later, and can be illustrated with the example of opisthokonts. Animals show a gradual relocation of all mitochondrial ribosomal protein genes to the host nucleus and elimination of the respective genes in the small mitochondrial genome. In *Choanozoa*, exemplified by *Monosiga brevicollis*, six mitochondrial genes encoding proteins homologous to the core set of plastid-encoded ribosomal genes (*rps*3, *rps*4, *rps*8, *rps*12, *rps*14, *rps*19) and three genes of the LSU subunit (*rpl*2, *rpl*14, *rpl*16; Lavrov et al. 2005) are present. The fungi, like the animals, have lost all r-protein genes from the mitochondrial genome (not shown in fig. 1) in some cases, with the exception of two core r-protein genes.

In the case of *Acanthamoeba castellanii*, the complete set of mitochondrial r-protein genes homologous to the core set of plastid-encoded ribosomal genes is expressed in the mitochondrion, with the exception of *rps*18 (Burger et al. 1995), which we did not detect in any mitochondrial genome sampled here. The same plastid core r-protein set is found in the mitochondria of two further members of the amoebozoa, *Polysphondylium palladium* and *Hartmannella vermiformis* (supplementary table S1, Supplementary Material online), as well as in *Dictyostelium citrinum* (Ogawa et al. 2000). On the other hand, *Physarum polycephalum* expresses only one ribosomal protein (Rps12) in the organelle (Takano et al. 2001). Because of additional losses of important genes such as *nad*2, *nad*4L, *nad*6, and *atp*8, coupled with the presence of several additional mitochondrial encoded Orfs not known from other mitochondrial genomes of Amoebozoa (in addition to a mF plasmid), *P. polycephalum* harbors a very unusual mitochondrial genome. A partial mitochondrial genome of a member of the Rhizaria, *Bigelowiella natans*, (AccNo.: HQ840955), indicates the presence of the complete plastid r-protein core set of ribosomal genes, with the exception of *rps*2, *rps*8, *rps*19, *rpl*2, and *rpl*36 within the partial sequence.

The “gold standard” of mitochondrial genomes is still that of *Reclinomonas americana* (Lang et al. 1997) that today is classified as a member of the Exacavata (Adl et al. 2005). Again, homologs of the entire plastid SSU r-protein core set (except Rps18 and Rpl36) are encoded by the mitochondrial genome. However, in the parasitic *Leishmania*, only *rps*12 was identified in the mitochondrial genome (AccNo.: NC_000894.1; Maslov et al. 1992). Homologs of the plastid core ribosomal set are encoded by many Stramenopiles (including Oomycetes) and Cryptophytes (Hauth et al. 2005; Kim et al. 2008), but in the Haptophyte *Emiliania huxleyi* in a reduced manner only (Sanchez Puerta et al. 2004; fig. 1).

Alveolates are known to have very small mitochondrial genomes. As exemplified by the Apicomplexan *Plasmodium falciparum*, no genes for r-proteins are encoded in the mtDNA and rRNAs are fragmented (Wilson and Williamson 1997; Feagin et al. 2012); the same might turn out to be true for peridinin-containing dinoflagellates. In the case of Ciliates, a common set of only two mitochondrial SSU r-proteins [Rps3, Rps4 (only Euplotes), Rps12, Rps13 and 14 (only in *Paramecium*)] and four LSU proteins (Rpl2, Rpl6, Rpl14, and Rpl16, see later) is encoded (de Graaf et al. 2009). Among the green plant lineages, *Marchantia polymorpha* and *Physcomitrella patens* mtDNA harbors the SSU r-protein core set (except Rps18), and Rpl2 is also encoded by the mitochondrion (Oda et al. 1992; Terasawa et al. 2007; fig. 1). Besides individual losses of one or two genes, this conclusion is also true for the Embryophytes (fig. 1). Rhodophytes, on the other hand, show evidence for a larger number of transfers of genes of the ribosomal core set from the mitochondrion to the cell nucleus (Leblanc et al. 1995; Ohta et al. 1998; Burger et al. 1999).

The pattern appears to be nearly identical in the case of mitochondrially encoded ribosomal genes homologous to the plastid core of the LSU subunit. Here, genes for Rpl2, Rpl14, and Rpl16 of the plastid core are detected in mitochondrial genomes (fig. 1). This is true for nearly all mitochondrial genomes encoding the plastid SSU r-protein core set, with the exception of some members of the green and red lines, Haptophytes, and, in the case of Rpl2, Cryptophytes. Thus, mitochondrially encoded r-proteins mirror the homologous core set found in plastids and genes encoding r-proteins are present in most mitochondria, with the exceptions of various parasites, the animals, and many fungi.

Very much in line with CoRR, the only mitochondria that have completely relinquished their genome are those that have relinquished their membrane-associated electron transport chain—hydrogenosomes and mitosomes (Müller et al. 2012)—and that have no need to keep a quinone pool in a state of redox poise (de Paula et al. 2012). Plastids entirely without genomes are, as yet, unknown.

### Ribosomal–Protein Genes in mtDNA and rRNA Lengths

If constraints imposed by the ribosomal assembly process underlie the retention of r-protein genes in mitochondria, why do some mitochondria express rRNAs but no r-proteins? Mitochondrial SSU and LSU rRNAs themselves might hold clues. We compared the length of mitochondrial rRNAs from organisms that encode the r-protein core set in mtDNA to those that do not. The SSU rRNA of mitochondria that encode the core set is at least 50% longer than that of animal mitochondria, which lack the core set. Similarly, the LSU rRNA of mitochondria that encode the core set is roughly twice as long as that found in animal mitochondria (supplementary table S1, Supplementary Material online). Thus, the loss of genes encoding the r-protein core set correlates with shortening of the mitochondrial rRNAs, at least in animals. Fungi are difficult to analyze with respect to r-proteins/rRNA lengths. On the one hand, identification of genes encoding r-proteins is complicated from biased fungal mitochondrial genomes as seen in the case of *rps*3 (Bullerwell et al. 2000); on the other hand, fungal mitochondrial genomes vary in the lengths of their rRNAs. However, at least in some studied cases, fungal SSU rRNAs share a conserved rRNA core region approximately equal in length to animal mitochondrial SSU rRNAs, and additional sequences, which inflate rRNA lengths, might be inserted into hotspots for insertions/deletions and map to the surface of the 30S ribosome (Barroso et al. 2003). This currently precludes a clear statement on the correlation between rRNA length and presence/absence of r-proteins in fungal mitochondria.

Clearly, the assembly of mitochondrial ribosomes can take place without coexpression of rRNA and r-protein genes in the same organelle. However, organellar rRNAs that approach the lengths of those found in *E. coli* appear to require r-protein genes to be expressed in the same compartment for proper assembly. Shorter mitochondrial rRNAs might have the intrinsic capacity to support ribosomal assembly without the help of r-proteins. It appears that it is not the ribosome itself, but rather the need for onsite expression for assembly that influences the presence or absence of genes encoding r-proteins in organellar genomes.

Although the opisthokonts (animals and fungi) possess the most derived mitochondria in terms of mt-DNA encoded r-proteins, the mitochondrial ribosome itself in these lineages is more protein-rich than in prokaryotes. In *E. coli* ribosomes, the RNA-to-protein mass ratio is 1:2, for yeast mitochondrial ribosomes it is 1:1, for bovine mitochondrial ribosomes it is 2:1 (Graack and Wittmann-Liebold 1998). Accordingly, *E. coli* ribosomes contain 53 proteins (Wittmann 1982) or 54 in a more recent genomic count (Yutin et al. 2012), whereas bovine mitochondrial ribosomes contain about 80 proteins (O'Brien 2003), and for yeast mitochondrial ribosomes, the value is closer to 90 (Graack and Wittmann-Liebold 1998). The majority of homologs for the standard prokaryotic r-proteins can be found encoded as nuclear genes in yeast or mammalian genomes, but they tend to be poorly conserved and many additional proteins lacking prokaryotic homologs have been recruited to the mitochondrial ribosome in these lineages (O'Brien 2003). The reasons for these recruitments are not known with certainty but have been suggested to involve compensation for the loss of many rRNA structural elements from mammalian and fungal lineages (O'Brien 2003).

### Looking Further: Eukaryotic r-Proteins

Plastid and mitochondrial genomes have undergone massively parallel evolution to encode the same functional set of genes in many independent lineages: genes for proteins of the electron transport chain of these bioenergetic organelles and genes for the ribosomes that are required to express them. The parallels in organelle gene content are, however, even stronger than previously recognized, because even the same set of r-proteins tends to be retained by plastid and mitochondrial genomes. This parallel retention of similar sets of r-proteins in plastid and mitochondrial genomes—the organelle r-protein core (Orpc; fig. 1)—clearly indicates the existence of a common selective pressure operating on both organelles. The location of r-proteins of the Orpc on the *E. coli* ribosome assembly map suggests that they have early and central roles in ribosome assembly. This might reflect hitherto unrecognized functional constraints underlying ribosomal assembly in organelles, constraints that tend to pin a specific subset of r-protein genes together with their cognate rRNAs in organellar genomes, thereby influencing the ability of r-protein coding genes to be relocated to the nucleus. In some lineages (animals), r-protein loss is accompanied by size reduction of rRNA 2-3-fold, in other lineages (fungi), insertions in rRNA seem to have compensated for loss of r-proteins from organellar genomes.

Does this principle apply to eukaryotic genomes as well? In eukaryotes, transcription and splicing are physically separated from translation by the nuclear envelope (Martin and Koonin 2006). In contrast to prokaryotes and organelles, eukaryotic 80S ribosome assembly is also separated from translation accordingly. Thus, synthesis of 80S ribosomes involves several steps in different subcellular compartments, and, at first glance, there seems to be no obvious reason to express r-proteins at a defined cellular localization. However, in some cases—eukaryotes with nucleomorphs—eukaryotic ribosomes are assembled in a different compartment from that in which the r-protein genes are localized (Curtis et al. 2012).

The Cryptophytes and Chlorarachniophytes evolved by secondary endosymbiosis and therefore harbor two phylogenetically different nuclei per cell (Maier et al. 2000; Curtis et al. 2012). Here, a eukaryotic cell either of green (Chlorarachniophyte) or red (Cryptophytes) algal origin became reduced in another eukaryotic cell, leading to a symbiont with a remnant eukaryotic cytoplasm, the periplastidal compartment (PPC) together with a vestigial nucleus, the nucleomorph (Hempel et al. 2007; Bolte et al. 2009). With less than 600 genes, nucleomorphs harbor highly reduced eukaryotic genomes (Douglas et al. 2001; Gilson et al. 2006; Lane et al. 2007; Curtis et al. 2012). However, the nucleomorph genes are expressed in the PPC via 80S ribosomes and factors missing for the functions of the PPC, which are not encoded by the nucleomorphs, are expected to be provided by the host (Curtis et al. 2012). In Cryptophytes and Chlorarachniophytes, some genes for r-proteins of the 80S ribosomes in the periplastidal compartment, where the nucleomorph resides, are encoded in the nucleomorph, while others are encoded in the host nucleus (Curtis et al. 2012).

Indeed, although nucleomorph genomes are highly reduced, encoding fewer than 600 proteins, one particularly conspicuous group of nucleomorph-encoded proteins in Cryptophytes and Chlorarachniophytes are the r-proteins for 80S ribosomes of the PPC. This is a eukaryotic version of the situation in plastids and mitochondria that have long rRNA subunits. A core set of genes can be defined as those encoding r-proteins, expressed in both Cryptophyte and Chlorarachniophyte nucleomorphs/PPCs (Curtis et al. 2012). This set comprises 21 SSU r-protein genes: ***rps*A** (S2), *rps*2, ***rps*3** (S3), *rps*3A, *rps*4, ***rps*5** (S7), *rps*6, rps8, ***rps*9** (S7), *rps*10, *rps*11, *rps*13, ***rps*14** (S11), ***rps*15** (S19), *rps*16, *rps*17, ***rps*23** (S12), *rps*26, *rps*27, *rps*27A, *rps*28. Strikingly, one-third of these, indicated in boldface type, are among the Orpc (the names of the corresponding prokaryotic/organelle homologs are given here in parentheses). For the 25 LSU r-protein genes of nucleomorphs, a slightly larger set is retained, and all members of the LSU Orpc are present: *rpl*3, *rpl*4, *rpl*5, *rpl*7A, ***rpl*8** (L2), *rpl*9, ***rpl*10** (L16), *rpl*10A, *rpl*11, *rpl*12, *rpl*13A, *rpl*14, *rpl*15, *rpl*17, *rpl*18A, *rpl*19, ***rpl*23** (L14), *rpl*24, *rpl*27, *rpl*27A, *rpl*30, *rpl*32, *rpl*34, *rpl*37A, *rpl*40 (supplementary table S2, Supplementary Material online).

Although the assembly of 80S ribosomes is a complicated process (Bernstein et al. 2004), several r-proteins have been shown to interact with the 18S rRNA (Granneman et al. 2010). Most of the nucleomorph SSU ribosomal core proteins are members of this group. Thus, nucleomorphs generally conform to the notion that ribosomal assembly helps to retain a specific set of r-protein genes in organelles, but nucleomorphs differ in one fundamental aspect from plastid and mitochondrial genomes. The flux of DNA from chloroplasts and mitochondria to the nucleus is a continuous and ongoing process, as evidenced by the finding that organelle DNA insertions are very common among sequenced eukaryotic genomes (Huang et al. 2005; Hazkani-Covo et al. 2010). By contrast, the flux of nucleomorph DNA has apparently come to a halt (Curtis et al. 2012), probably because, for Cryptophytes, there is only one nucleomorph per cell; hence, there are few or no “leftover” nucleomorph chromosomes that could be transferred to the nucleus.

**Fig. 3.—:**
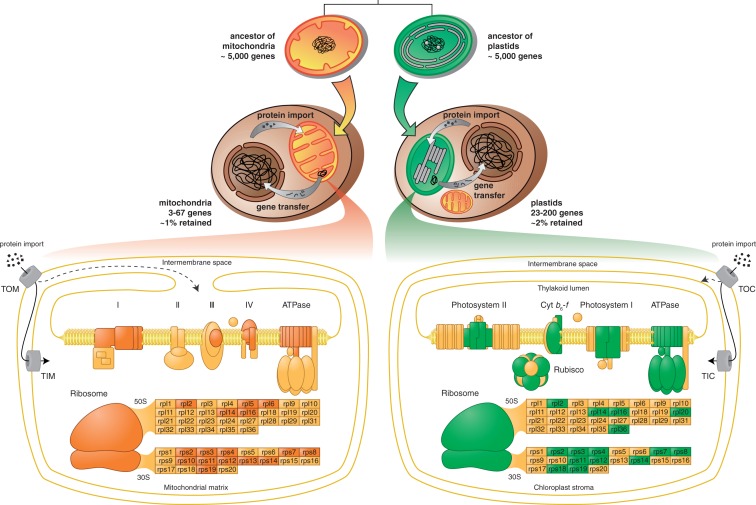
Parallel evolution of mitochondria and plastids. The ancestors of both organelles were prokaryotes with nonreduced genomes encoding around 5,000 genes. During the course of endoymbiosis, genes were transferred from organellar host nuclear genomes, and the corresponding gene products were imported back to the organelles. The initial genome size of several thousand dwindled to 3–67 in mitochondria and 23–200 in plastids. The lower part of the figure shows the parallels between the retained genes in mitochondria and plastids (oxidative phosphorylation, photosynthesis, and ribosomes). Organellar-encoded genes are colored brown for mitochondria and green for plastids. Schemes for oxidative phosphorylation and photosynthesis were adapted from Allen (2003).

Thus, the gene content that we see in cryptophytic nucleomorphs has much more of the character a frozen accident than the gene content of plastids and mitochondria, where gene transfer is constant and ongoing, begging the question of why any genes are retained in organelles at all. CoRR provides the answer (Allen 2003); it furthermore accounts for which genes are retained (those for electron transport chain components and ribosomes to synthesize them), in addition to accounting for why organelle genomes are relinquished when membrane-associated electron transport is lost. Our present findings add to this view by showing that, among r-proteins, there exist additional selective pressures that lead to the preferential retention of their genes. Selection has thus resulted in the retention of a common set of genes in organelles, for electron transport chain components, and for the organelle r-protein core, as summarized in figure 3. These gene products have been conserved among plastid lineages, among mitochondrial lineages, and across the plastid–mitochondrial boundary. This might be biology’s most striking case of convergent evolution.

## Conclusions

The endosymbiotic cyanobacteria of *Azolla* (Ran et al. 2010) or the *Paulinella* endosymbiont (Nowack et al. 2008) tend to retain the full set of r-proteins, with only Rpl25 (*Paulinella chromatophora*) and RpS1 (*Nostoc azollae*) having been lost in comparison with the *E. coli* set. Endosymbiotic bacteria such as *Buchnera* (Shigenobu et al. 2000), *Wolbachia* (Wu et al. 2004), and others do, however, tend to lose one or the other r-protein gene (McCutcheon 2010). Such attrition of r-protein gene content is shown in figure 1, where it is seen that in the albeit small sample of endosymbionts considered here, the organelle core set remains intact. Because endosymbionts cannot import nuclear-encoded precursor proteins in the same way that plastids and mitochondria can, they retain their genes for r-proteins somewhat more tenaciously than organelles do. This tenacity is best exemplified by *Tremblaya*, which has retained 44 r-proteins even though only 121 proteins are encoded in the whole *Tremblaya* genome (McCutcheon and von Dohlen 2011). Naturally minimized systems such as plastids, mitochondria, and the reduced nuclei of some secondary symbiotic plastids provide a window into the evolutionary process. For bioenergetic organelles, we see a different kind of reductive evolution from that in endosymbiotic bacteria such as *Buchnera* (Moran 2007). In endosymbiotic bacteria, reduction leads to genomes that express genes and that lose genes in such a way that the loss can be compensated by the import of small molecular weight metabolites across the plasma membrane. A possible exception is *Tremblaya* that harbors no protein-coding genes for amino acyl tRNA synthetases; these are probably provided by lysed γ-proteobacterial endosymbionts that live within these endosymbiotic β-proteobacteria (Husnik et al. 2013).

In bioenergetic organelles (plastids and mitochondria), reduction leads to genomes that can express genes and that lose genes in such a way that the loss can be compensated either by the import of small molecular weight metabolites across the inner membranes or by import of nuclear-encoded proteins. Because of the capacity for protein import, organelles could, in principle, lose their genome altogether, as has happened in the case of hydrogenosomes and mitosomes (de Paula et al. 2012). However, in a case of massive convergence in many independent lineages, plastids and mitochondria have evolved to retain genes for the proteins of the electron transport chain and for the ribosome. Plastid and mitochondrial genomes have been intensely studied; it is therefore all the more surprising that it has gone so far unnoticed that they have furthermore converged on the same set of r-proteins—Rps2, Rps3, Rps4, Rps7, Rps8, Rps11, Rps12, Rps14, and Rps19 in the 30S subunit and Rpl2, Rpl14, and Rpl16 in the 50S subunit—starting from 54 to 55 r-proteins in the typical cyanobacterial and proteobacterial ribosomes from which ribosomes of plastids and mitochondria arise. This indicates the presence of strong selective pressure to maintain the genes for these proteins in the organelle—for reasons of ribosome assembly, we suggest. When the organelle rRNA length falls below a threshold of approximately 1,300 nt (SSU) or 2,100 nt (LSU), as has happened in vertebrate mitochondrial DNA, it appears that a functional threshold is crossed, removing the selective pressure to retain the organelle ribosomal protein core, and all r-proteins are then apparently freed to become encoded in the nucleus. But even when all ribosomal protein genes have migrated to the nucleus, quinone-dependent electron transport in bioenergetic membranes anchors genes for components of the electron transport chain in the organelle. When quinone-dependent electron transport is lost from the organelle, the genome is lost as well.

The periplastidal compartment of Chlorarachniophytes and Cryptophytes, where the nucleomorph resides, is not a bioenergetic organelle. It is therefore fully in line with the CoRR hypothesis that nucleomorphs do not preferentially encode components of electron transport chains (only about 4% of their proteins are targeted to the plastid); instead, they encode more or less typical cytoplasmic proteins, involved in folding, mitosis, and the like, including a major complement of genes for the synthesis of 80S ribosomes (Maier et al. 2000; Douglas et al. 2001; Gilson et al. 2006; Lane et al. 2007). As in plastids and mitochondria, a colocalization of genes for many r-proteins and rRNA is found in nucleomorphs. In both nucleomorph-containing protist groups, the nucleomorph-specific 18S rRNA is longer than the host copies, and indeed, the intersection of nuclemorph-encoded genes for r-proteins in the two lineages is very high, contrary to most of the rest of the nucleomorph-encoded proteome. Thus, it appears that the same constraint is operating on the ribosomes of naturally reduced genomes in organelles of both prokaryotic and eukaryotic origin.

## Supplementary Material

Supplementary tables S1 and S2 are available at *Genome Biology and Evolution* online (http://www.gbe.oxfordjournals.org/).

Supplementary Data
