# Supplementary material for: Massively Convergent Evolution for Ribosomal Protein Gene Content in Plastid and Mitochondrial Genomes
Source: Genome Biol Evol. 2013 Nov 19;5(12):2318–29. doi: 10.1093/gbe/evt181 (PMC3879969; doi:10.1093/gbe/evt181)
Supplement: Supplementary Data [file supp_5_12_2318__index.html]

Massively convergent evolution for ribosomal protein gene content in plastid and mitochondrial genomes — Massively Convergent Evolution for Ribosomal Protein Gene Content in Plastid and Mitochondrial Genomes — Supplementary Data 

# Massively Convergent Evolution for Ribosomal Protein Gene Content in Plastid and Mitochondrial Genomes

## Supplementary Data

files

**Files in this Data Supplement:**

- Supplementary Data - docx file
- Supplementary Data - docx file
