# Supplementary material for: Massively Convergent Evolution for Ribosomal Protein Gene Content in Plastid and Mitochondrial Genomes
Source: Genome Biol Evol. 2013 Nov 19;5(12):2318–29. doi: 10.1093/gbe/evt181 (PMC3879969; doi:10.1093/gbe/evt181)
Supplement: Supplementary Data [file supp_evt181_Supplementary_Table_1_revised.docx]

**Supplementary Table 1**: Organisms, Accession numbers and length of analyzed genomes. Hodgkinia and Carsonella were adopted from McCutcheon 2010. * partial genome.

| **Organism** | **Acc.No.:** | **l-rRNA**  **length** | **s-rRNA length** |
| --- | --- | --- | --- |
| *Rhodobacter sphaeroides* 2.4.1 | NC_007493 | 2882 | 1464 |
| *Anabena variabilis* | NC_007413 | 2829 | 1487 |
| *Candidatus Tremblaya princeps* PCVAL | NC_017293 | 2876 | 1520 |
| *Candidatus Riesia pediculicola* USDA | NC_014109 | 2881 | 1559 |
| *Wigglesworthia glossinidia* (endosymbiont of *Glossina brevipalpis*) | NC_004344 | 2921 | 1550 |
| *Lawsonia intracellularis* PHE/MN1-00 | NC_008011 | n.d. | n.d. |
| **Chromatophore** | | | |
| *Paulinella chromatophora* | NC_011087 | 2871 | 1444 |
| **Chloroplast** | | | |
| *Euglena gracilis* | NC_001603 | 2877 | 1492 |
| *Astasia longa* | NC_002652 | 3134 | 1452 |
| *Bigelowiella natans* | NC_008408 | 2889 | 1435 |
| *Alveolata* sp. CCMP3155 | NC_014345 | 2765 | 1492 |
| *Chromera velia* | NC_014340 | 3058 | 1585 |
| *Babesia bovis* T2Bo | NC_011395 | n.d. | n.d. |
| *Eimeria tenella* strain Penn State | NC_004823 | 2745 | 1509 |
| *Theileria parva* strain Muguga | NC_007758 | 2775 | 1507 |
| *Toxoplasma gondii* RH | NC_001799 | 2686 | 1499 |
| *Plasmodium falciparum* HB3 | NC_017928 | n.d. | n.d. |
| *Emiliania huxleyi* | NC_007288 | 2872 | 1484 |
| *Thalassiosira pseudonana* | NC_008589 | 2891 | 1485 |
| *Odontella sinensis* | NC_001713 | 2891 | 1485 |
| *Cryptomonas paramecium* | NC_013703 | 2778 | 1492 |
| *Guillardia theta* | NC_000926 | 2888 | 1487 |
| *Cyanophora paradoxa* | NC_001675 | 2926 | 1495 |
| *Cyanidium caldarium* | NC_001840 | 2918 | 1527 |
| *Cyanidioschyzon merolae* | NC_004799 | 2853 | 1425 |
| *Porphyra purpurea* | NC_000925 | 2888 | 1496 |
| *Mesostigma viride* | NC_002186 | 2911 | 1511 |
| *Chara vulgaris* | NC_008097 | 2942 | 1515 |
| *Chaetosphaeridium globosum* | NC_004115 | 2875 | 1492 |
| *Nephroselmis olivacea* | NC_000927 | 2941 | 1514 |
| *Micromonas* sp. RCC299 | NC_012575 | 2056 | 1499 |
| *Chlorella vulgaris* | NC_001865 | 2994 | 1494 |
| *Helicosporidium* sp. ex Simulium jonesii | NC_008100 | 2862 | 1482 |
| *Equisetum arvense* | NC_014699 | 2798 | 1496 |
| *Isoetes flaccida* | NC_014675 | 3011 | 1505 |
| *Physcomitrella patens* | NC_005087 | 2817 | 1498 |
| *Marchantia* | NC_001319 | 2811 | 1496 |
| *Pinus thunbergii* | NC_001631 | 2803 | 1491 |
| *Epifagus virginiana* | NC_001568 | 2804 | 1492 |
| *Zea mays* | NC_001666 | 2884 | 1491 |
| *Oryza sativa* japonica group | NC_001320 | 2884 | 1491 |
| *Nicotiana tabacum* | NC_001879 | 2810 | 1491 |
| *Oenothera elata* | NC_002693 | 2835 | 1492 |
| *Cuscuta reflexa* | NC_009766 | 2809 | 1490 |
| *Cuscuta gronovii* | NC_009765 | 2835 | 1491 |
| *Cuscuta exaltata* | NC_009963 | 2809 | 1490 |
| *Cuscuta obtusiflora* | NC_009949 | 2826 | 1491 |
| **Mitochondria** | | | |
| *Euplotes minuta* | GQ903130 | 3053 | 2256 |
| *Euplotes crassus* | GQ903131 | 2232 | 2327 |
| *Tetrahymena pyriformis* | NC_000862 | 2315/280 | 1407/208 |
| *Paramecium aurelia* | NC_001324 | 2279/289 | 1477/204 |
| *Paramecium caudatum* | NC_014262 | 2339/291 | 1447/204 |
| *Thalassiosira pseudonana* | NC_007405 | 2803 | 1584 |
| *Synedra acus* | NC_013710 | 2723 | 1550 |
| *Phaeodactylum tricornutum* | NC_016739 | 2751 | 1556 |
| *Blastocystis* sp. DMP/02-328 | NC_011212 | 2655 | 1569 |
| *Chattonella marina* | NC_013837 | 2696 | 1519 |
| *Cafeteria roenbergensis* | NC_000946 | 2595 | 1662 |
| *Chrysodidymus synuroideus* | NC_002174 | 2586 | 1579 |
| *Desmarestia viridis* | NC_007684 | 2696 | 1541 |
| *Dictyota dichotoma* | NC_007685 | 2642 | 1557 |
| *Fucus vesiculosus* | NC_007683 | 2667 | 1520 |
| *Pylaiella littoralis* | NC_003055 | 2707 | 1519 |
| *Laminaria digitata* | NC_004024 | 2731 | 1535 |
| *Ochromonas danica* | NC_002571 | 2591 | 1563 |
| *Proteromonas lacertae* | NC_014338 | 2689 | 1527 |
| *Pythium ultimum* | NC_014280 | 2655 | 1511 |
| *Saprolegnia ferax* | NC_014280 | 2868 | 1529 |
| *Phytophthora infestans* | NC_002387 | 2654 | 1503 |
| *Emiliania huxleyi* | NC_005332 | 2688 | 1548 |
| *Rhodomonas salina* | NC_002572 | 2663 | 1483 |
| *Cyanophora paradoxa* | NC_017836 | 2659 | 1486 |
| *Glaucocystis nostochinearum* | NC_015117 | 2645 | 1393 |
| *Chondrus crispus* | NC_001677 | 2583 | 1376 |
| *Porphyra purpurea* | NC_002007 | 2588 | 1407 |
| *Cyanidioschyzon merolae* | NC_000887 | 2728 | 1542 |
| *Oltmannsiellopsis viridis* | NC_008256 | 2669 | 1539 |
| *Ostreococcus tauri* | NC_008290 | 2586 | 718/754 |
| *Prototheca wickerhamii* | NC_001613 | 3009 | 1674 |
| *Pseudendoclonium akinetum* | NC_005926 | 2776 | 1424 |
| *Nephroselmis olivacea* | NC_008239 | 2760 | 1509 |
| *Micromonas* sp. RCC299 | NC_008239 | 2303 | 693/765 |
| *Mesostigma viride* | NC_008240 | 2843 | 1558 |
| *Chara vulgaris* | NC_005255 | 2823 | 1572 |
| *Chaetosphaeridium globosum* | NC_004118 | 2693 | 1491 |
| *Pleurozia purpurea* | NC_013444 | 2807 | 1975 |
| *Marchantia polymorpha* | NC_001660 | 2799 | 1975 |
| *Physcomitrella patens* | NC_007945 | 2946 | 1587 |
| *Arabidopsis thaliana* | NC_001284 | 2568 | 1935 |
| *Nicotiana tabacum* | NC_006581 | 3406 | 1902 |
| *Malawimonas jakobiformis* | NC_002553 | 2731 | 1573 |
| *Naegleria gruberi* | NC_002573 | 2673 | 1579 |
| *Acanthamoeba castellanii* | NC_001637 | 2719 | 1541 |
| *Dictyostelium citrinum* | NC_007787 | 2874 | 1540 |
| *Hartmannella vermiformis* | NC_013986 | 2760 | 1508 |
| *Polysphondylium pallidum* | NC_006862 | 2764 | 1516 |
| *Reclinomonas americana* | NC_001823 | 2751 | 1595 |
| *Monosiga brevicollis* | NC_004309 | 2878 | 1596 |
| Bigelowiella natans | HQ840955* | 2434 | 1347 |
| Homo sapiens | NC_012920 | 1558 | 953 |
| Bos taurus | NC_006853 | 1570 | 954 |
| Carassius auratus | NC_002079 | 1680 | 953 |
| Apis mellifera ligustica | NC_001566 | 1370 | 785 |
| Tribolium castaneum | NC_003081 | 1279 | 773 |
