# Supplementary material for: Massively Convergent Evolution for Ribosomal Protein Gene Content in Plastid and Mitochondrial Genomes
Source: Genome Biol Evol. 2013 Nov 19;5(12):2318–29. doi: 10.1093/gbe/evt181 (PMC3879969; doi:10.1093/gbe/evt181)
Supplement: Supplementary Data [file supp_evt181_Supplementary_Table_2.docx]

**Table S2.** Ribosomal proteins encoded on the nucleomorph of three cryptophytes and one chlorarachniophyte. Proteins encoded on all four genomes are shown in bold, Orpc proteins are highlighted in green.

|  | **Cryptophyta** | | | **Chlorarachniophyta** |  |
| --- | --- | --- | --- | --- | --- |
| eukaryotic ribosomal protein | *Guillardia theta* | *Hemiselmis andersenii* | *Cryptomonas paramecium* | *Bigelowiella natans* | prokaryotic homolog |
| **rpSA** | **rsp4** | **rsp4** | **rsp4** | **rps0** | rpS2 |
| **rpS2** | **rpS2** | **rps2** | **rps2** | **rps2** | rpS5 |
| **rpS3** | **rpS3** | **rps3** | **rps3** | **rps3** | rpS3 |
| **rpS3A** | **rpS3A** | **rps3a** | **rps3a** | **rps3a** |  |
| **rpS4** | **rpS4** | **rps4** | **rps4** | **rps4** |  |
| **rpS5** | **rpS5** | **rps5** | **rps5** | **rps5** | rpS7 |
| **rpS6** | **rpS6** | **rps6** | **rps6** | **rps6** |  |
| rpS7 |  |  |  | rps7 |  |
| **rpS8** | **rpS8** | **rps8** | **rps8** | **rps8** |  |
| **rpS9** | **rpS9(S7)** | **rps9** | **rps9** | **rps9** | rpS4 |
| **rpS10** | **rpS10B** | **rps10B** | **rps10B** | **rps10B** |  |
| **rpS11** | **rpS11** | **rps11** | **rps11** | **rps11** | rpS17 |
| rpS12 |  |  |  | rpS12 |  |
| **rpS13** | **rpS13** | **rps13** | **rps13** | **rps13** | rpS15 |
| **rpS14** | **rpS14** | **rps14** | **rps14** | **rps14** | rpS11 |
| **rpS15** | **rpS15** | **rps15** | **rps15** | **rps15** | rpS19 |
| rpS15A | rpS15A | rps15A | rps15A |  | rpS8 |
| **rpS16** | **rpS16** | **rps16** | **rps16** | **rps16** | rpS9 |
| **rpS17** | **rpS17** | **rps17** | **rps17** | **rps17E** |  |
| rpS18 |  |  |  | rps18 | rpS13 |
| rpS19 | rpS19 | rps19 | rps19 |  |  |
| rpS20 | rpS20 | rps20 | rps20 |  | rpS10 |
| rpS21 | rpS21 | rps21 |  |  |  |
| **rpS23** | **rpS23** | **rps23** | **rps23** | **rps23** | rpS12 |
| rpS24 | rpS24 | rps24 | rps24 |  |  |
| rpS25 | rpS25 | rps25 | rps25 |  |  |
| **rpS26** | **rpS26** | **rps26** | **rps26** | **rps26** |  |
| **rpS27** | **rpS27** | **rps27** | **rps27** | **rps27** |  |
| **rpS27A** | **rpS27A** | **rps27A** | **rps27A** | **rps27A** |  |
| **rpS28** | **rpS28** | **rps28** | **rps28** | **rps28** |  |
| rpS29 | rpS29 | rps29 | rps29A |  | rpS14 |
| rpS30 |  | rps30 | rps30 | rps30 |  |
| **rpL3** | **rplL3** | **rpl3** | **rpl3** | **rpl3** | rpL3 |
| **rpL4** | **rpL4/L1** | **rpl4/1** | **rpl4/1** | **rpl4** |  |
| **rpL5** | **rpL5** | **rpl5** | **rpl5** | **rpl5** | rpL18 |
| rpL6 | rpL6B | rpl6B | rpl6B |  |  |
| rpL7 | rpL7 | rpl7 | rpl7 |  | rpL30 |
| **rpL7A** | **rpL7A** | **rpl7A** | **rpl7A** | **rpl7Ae** |  |
| **rpL8** | **rpL8** | **rpl8** | **rpl8** | **rpl8** | rpL2 |
| **rpL9** | **rpL9** | **rpl9** | **rpl9** | **rpl9** | rpL6 |
| **rpL10** | **rpL10** | **rpl10** | **rpl10** | **rpl10/e** | rpL16 |
| **rpL10A** | **rpL10A** | **rpl10A** | **rpl10A** | **rpl10A** | rpL1 |
| **rpL11** | **rpL11B** | **rpl11B** | **rpl11B** | **rpl11** | rpL5 |
| **rpL12** | **rpL12** | **rpl12** | **rpl12** | **rpl12 *** | rpL11 |
| rpL13 | rpL13 | rpl13 | rpl13 |  |  |
| **rpL13A** | **rpL13A** | **rpl13A** | **rpl13A** | **rpl13A** | rpL13 |
| **rpL14** | **rpL14** | **rpl14** | **rpl14** | **rpl14A** |  |
| **rpL15** | **rpL15** | **rpl15** | **rpl15** | **rpl15** |  |
| **rpL17** | **rpL17** | **rpl17** | **rpl17** | **rpl17** | rpL22 |
| rpL18 | rpL18 | rpl18 | rpl18 |  |  |
| **rpL18A** | **rpL18A** | **rpl18A** | **rpl18A** | **rpl18A** |  |
| **rpL19** | **rpL19** | **rpl19** | **rpl19** | **rpl19** |  |
| rpL21 | rpL21 | rpl21 | rpl21 |  |  |
| **rpL23** | **rpL23** | **rpl23** | **rpl23** | **rpl23** | rpL14 |
| rpL23A | rpL23A | rpl23A | rpl23A |  | rpL23 |
| **rpL24** | **rpL24** | **rpl24** | **rpl24** | **rpl24** |  |
| rpL26 | rpL26 | rpl26 | rpl26 |  | rpL24 |
| **rpL27** | **rpL27** | **rpl27** | **rpl27** | **rpl27** |  |
| **rpL27A** | **rpL27A** | **rpl27A** | **rpl27A** | **rpl27A** | rpL15 |
| **rpL30** | **rpL30** | **rpl30** | **rpl30** | **rpl30** |  |
| rpL31 | rpL31 | rpl31 | rpl31 |  |  |
| **rpL32** | **rpL32** | **rpl32** | **rpl32** | **rpl32** |  |
| **rpL34** | **rpL34** | **rpl34** | **rpl34** | **rpl34** |  |
| rpL35A | rpL35A | rpL35A | rpL35A |  |  |
| rpL36 | rpL36 | rpL36 | rpL36 |  |  |
| rpL36A |  |  |  | rpl44 |  |
| **rpL37A** | **rpL37A** | **rpL37A** | **rpL37A** | **rpL37Ae** |  |
| **rpL40** | **rpL40** | **rpl40** | **rpl40** | **rpl40** |  |
| rpLP0 | rla0/P0 | rla0/P0 | rla0/P1 |  | rpL10 |
| rpLP1 | rla1/P1 |  |  |  | rpL7/L12 |
